# Digitally transformed home office impacts on job satisfaction, job stress and job productivity. COVID-19 findings

**DOI:** 10.1371/journal.pone.0265131

**Published:** 2022-03-10

**Authors:** Ludivine Martin, Laetitia Hauret, Chantal Fuhrer

**Affiliations:** 1 LISER - Luxembourg Institute of Socio-Economic Research, Esch-sur-Alzette, Luxembourg; 2 CREM - Center for Research in Economics and Management (UMR CNRS 6211), Rennes, France; 3 University of La Réunion, La Réunion, France; 4 Institut d’Administration des Entreprises, Grenoble, France; Institute for Advanced Sustainability Studies, GERMANY

## Abstract

In these times of successive lockdown periods due to the health crisis induced by COVID-19, this paper investigates how the usages of collaborative and communication digital tools (groupware, workflow, instant messaging and web conference) are related to the evolution of teleworkers’ subjective well-being (job satisfaction, job stress) and job productivity comparing during and before the first lockdown in spring 2020. Using a sample of 438 employees working for firms located in Luxembourg, this analysis enables, first, to highlight different profiles of teleworkers regarding the evolution of usages of these tools during the lockdown compared to before and the frequency of use during. Second, the analysis highlights that these profiles are linked to the evolution of job satisfaction, job stress and job productivity. Our main results show that (1) the profile that generates an increase in job productivity is the one with a combined mastered daily or weekly use of all of the four studied digital tools but at the expense of job satisfaction. On the contrary, (2) the use of the four digital tools both before and during the lockdown, associated with an increase in the frequency of use, appears to generate too much information flow to deal with and teleworkers may suffer from information overload that increases their stress and reduces their job satisfaction and job productivity. (3) The habit of using the four tools on a daily basis before the lockdown appears to protect teleworkers from most of the adverse effects, except for an increase in their job stress. Our results have theoretical and managerial implications for the future of the digitally transformed home office.

## 1 Introduction

The numerous impacts of COVID-19 and particularly the role of information systems and technologies in this crisis are of research interest [1–6].

Successive lockdowns have shaken everybody’s life [7]. At the work sphere, they have shaken the relationship to work, in time, space and form, for a large part of workers. The main adaptation to this shock was home office [8–10]. The definition of home office adopted for this research involves working from home, away from the traditional office, with the help of computers or other digital facilities to maintain a link to the office [11–14]. The concept of home office is often associated with other close concepts including telecommuting, working from home, teleworking, mobile work, flexiplace, satellite office, detached units, distance meetings, or virtual organization [15, 16].

In the late 1970s and the 1980s, home office was perceived as the work arrangement of the future [17]. As underlined by [18], it becomes increasingly feasible thanks to advances in Information and Communication Technologies (ICTs). Advantages and disadvantages of home office are numerous at the organizational and individual levels. For employers, the practice helps productivity, profitability and flexibility [14, 19–21]. It is also associated with a decrease of employees’ absenteeism and turnover [17, 22]. Some authors highlight the improvement in remote collaborations [23]. Nevertheless, most authors highlight the lack of cooperation and team spirit as disadvantages of home office [12, 14, 20, 24]. Other disadvantages of home office at the employer level, include managerial, administrative, and legislative problems to implement the practice [20]. For instance, the reduced supervisor control leads to decreased timeliness of work completion and questions the risk of employees’ cyberslacking [14, 16]. For employees, the flexibility offered by the home office practice in the allocation of time and energies participates to better job satisfaction and quality of life [14, 20, 25–27]. Home office offers also autonomy, greater concentration and fewer interruptions [28, 29]. Another advantage for home office is economic, i.e. lower housing and commuting cost, travel cost [19, 20]. Some authors show greater commitment, work effort and performance associated with the practice [29, 30] when some others highlight the lack of contact and reduced collaboration opportunities, social problems and isolation [12, 20, 31, 32]. Another disadvantage is lower salary growth and professional advancement of teleworkers [17, 33, 34].

Before the COVID-19 lockdown periods, the corporate use of home office was usually occasional [18, 35]. However, during the lockdown, working full-time at home, when it was possible, became a mandatory working mode. Home office was indeed sudden, not an option, and not anticipated [36]. In US, [37] observe that 5% of all full workdays were supplied from home before the lockdown with an increase to 50% during May-October 2020. In UK, 4.7% of those in employment reported working mainly at home in 2019 and it roses to 43% in April 2020 [38]. In France, 3% of employees worked remotely in 2017 [39] and it increases to 32% during the lockdown with 75% of teleworkers who discovered this practice for the first time [40]. In Luxembourg, 20% of resident employees worked remotely over the period 2015–2019 and it increases to 52% during the lockdown with 74% of teleworkers who experienced it during this period [41].

In the context of a widespread home office practice induced by the lockdown periods, studies highlight a strong heterogeneity and mixed results regarding teleworkers’ job well-being and job productivity while having a happier workforce is profitable for firms as it was shown to increase establishment-level productivity [42]. Regarding teleworkers’ job well-being, some empirical results reveal negative effects such as mental health troubles [38] while some others underline that teleworkers are more autonomous, more engaged and experience fewer negative emotions [43]. For teleworkers’ job productivity, once again some studies report a positive impact of the lockdown [37, 44, 45] while others reveal negative or absence of job productivity evolution [46, 47]. The role played by digital tools is acknowledge in some analyses [46, 48, 49]. They underline that a high-quality digital environment reduces the lack of social interactions with co-workers during lockdown periods. Nevertheless, the effective role played by digital tools use is largely neglected.

Therefore, the objective of this article is to address this gap by examining the following research question: under what conditions of digital tools use did the lockdown increase well-being at work and/or job productivity among teleworkers?

To tackle this research question, we draw from the Adaptive Structuration Theory [50] to understand the workers’ appropriation of a digitally transformed home office. We hypothesize that there are specific digital contexts of home office enablers that are associated with higher levels of job satisfaction and job productivity, and barriers that are associated with more job stress. We consider in this paper the evolution of teleworkers’ self-reported perceived job satisfaction, job stress and job productivity during the lockdown compared to before. We test our hypotheses on survey data obtained from the ‘home office’ module of the COVID-19 socio-economic impacts (SEI) survey conducted between end of May and early July 2020 on around 450 employees working in Luxembourg. In Luxembourg, a general lockdown was applied between March 16 and May 3, 2020. As cross-border workers represent around 48% of the workforce employed in Luxembourg, bilateral fiscal agreements were concluded with bordering countries in order to facilitate home office.

The article is structured as follows. In the next section, we present our theoretical background and literature review. Then we describe the methods we adopted in this research and the data studied. After, we present and discuss the results. The last section discusses the contributions of the paper to the literature as well as its managerial implications.

## 2 Theoretical background and existing empirical evidence

We first present perspectives from the Adaptive Structuration Theory to understand individual’s appropriation of a digitally transformed home office and under what conditions they judge it participates to their job satisfaction, their job stress and/or their job productivity. Second, we review prior research on the evolution of teleworkers’ well-being at work and job productivity during the lockdown. Third, as some previous researches highlight the importance of digital knowledge and Information and Communication Technologies (ICT) quality of equipment on these evolutions, we present empirical evidence on the role the usages of digital tools can play to improve well-being and productivity at work.

### 2.1 Adaptive Structuration Theory (AST)

Our theoretical background to better capture under what conditions the digitally transformed home office can improve teleworkers’ job well-being and job productivity is based on the Adaptive Structuration Theory (AST) developed by [50]. This theory looks at how the technology is designed and how the technology is used and interpreted by the end user. Despite it was originally a group level analysis theory, we argue it is also relevant for an individual level of analysis. It goes beyond the technocentric view of technology use [51] in which the technology has a determining role in predicting changes inside companies and the humancentric perspective in which individuals’ interpretations and agency are only considered [52]. In the AST perspective, individuals, and organizations using technology for their work dynamically create perceptions about the role and utility of the technology, and how it can be applied to their activities. These perceptions can vary widely across individuals and organizations. They influence how digital tools are used and appraised and consequently mediate their impact on individuals and organizations outcomes.

From this perspective, the digital tools have characteristics that are constraining, i.e. they are not modified by the user once they are implemented. The technology choice by user consider the ‘spirit’ of the technology [50]. Towards digital tools, which use remains optional, the user can adopt various behaviors: total rejection, minimal use or intensive use. Total rejection means that user prefers to keep his old way of working; the technology thus is not adopted. Minimal use signifies that the user limits himself to common uses, generally those for which he has received initial training. Intensive use occurs when the user constantly seeks to improve his mastery of the tool. Appropriation refers to the process by which the user integrates, to varying degrees, the use of the tool into his operating mode and possibly can, and alone, make this operating mode evolve according to the properties of the tool he discovers. The effective appropriation of technology needs the combination of three conditions: a minimum mastery of the tool, the integration of the use of the tool in daily practices, and the possibility of the emergence of an innovative use of the tool. These three dimensions are involved in the appropriation (or assimilation) of a technology. The first relates to the technological characteristics of the tool, as perceived by the user. For example, perceived usefulness or perceived ease of use. The second is related to the context in which the technology is used. For instance, the existence of training or support for users. The third is related to individual characteristics: age, gender, socio-professional category, sensitivity to technology, change, pressure, etc.

Therefore, the characteristics of the digital environment supporting the home office practice, as well as the characteristics of workers, may have an effect on its appropriation. In such way, we consider both technical and social ‘artifact’ of home office [53]. These two sets of features are of equal importance and specific combinations of both are often key to understanding and anticipating consequences. For example, individual characteristics, job characteristics, workplace characteristics probably interfere when using groupware, workflow, instant messaging and/or web conference. The worker appraisal probably differs towards his perceived job satisfaction, stress and productivity.

However, the COVID-19 crisis questions this traditional vision of appropriation. First, home office and using digital tools unknown by part of teleworkers during lockdown periods was not optional. Total rejection was thus impossible because the solution of home office was imposed by the sanitary situation and requested by governments. Second, the time devoted to learning how to home office was null or very short, at least at the very beginning of the first lockdown. The home office solution was sudden and unanticipated both by the employer and by the employee. During this period of imposed distancing, home office was subject to two forms of appropriation: an appropriation corresponding to the sharing out pattern [54] where the tool is seen as a means of exchange with others, and an appropriation of continuity of activity, centered on the activities to be maintained. In 2020, the constraint to adopt new digital tools during the lockdown periods was external and put managers and employees in the same boat, which may ease the transition [55]. [56] show indeed that individuals are willing to make more efforts easily, and even accept a more unequal sharing of the benefits achieved, if constraints come from people outside their group. For employees, the lockdown is itself an external constraint, like a diffuse scapegoat and to which employees cannot blame. In other circumstances, employees might have been reluctant to work in a digitally transformed work environment while feeling a constraint on the part of their management.

### 2.2 Evolution of job well-being and productivity feelings between before and during the lockdown among teleworkers

#### 2.2.1 Well-being at work evolution in the context of a widespread home office practice

Research on direct effects of home office on job satisfaction and job stress remains scarce in the context of lockdown periods. [57] regarding various well-being indicators such as negative affect (depression, anxiety, worry, and lack of interest in daily activities) or happiness at work, highlight heterogeneity between countries and variation all along 2020 in parallel to changes in sanitary restrictions. [38] use data from the ‘Understanding Society’ COVID-19 survey (April, May and June 2020). Regarding job quality, they underline that home office is linked with mental health issue but the phenomenon decreases with the time or with the move back to traditional places of work. [43] assess fifteen workers’ well-being and performance outcomes collected from 621 full-time workers assessed before (from November 2019 to February 2020) and during (May-June 2020) the COVID-19 pandemic. They reveal that the majority of employees’ well-being measures are not adversely affected. Teleworkers feel more engaged and autonomous, experience fewer negative emotions and feel more connected to their organizations. Some other studies underline that the extensive use of home office negatively influences job satisfaction due to less interactions between co-workers [49]. Therefore, it is interesting to examine more precisely the evolution of satisfaction and more generally of well-being at home office, before and after the first lockdown. As the study of well-being is closely linked to the study of performance [42], a complementary topic is to examine the evolution of productivity in these same contexts.

#### 2.2.2 Job productivity evolution in the context of widespread home office practice

Previous research on the evolution of teleworkers’ subjective job productivity between before and during the COVID-19 induced lockdown periods reveals mixed result and heterogeneity among teleworkers. Some papers highlight a positive evolution of job productivity. For example, [44], by surveying 592 Amazon MTurk respondents, conclude that during the lockdown respondents saw an increase in their productivity and of their subordinates’ productivity compared as before. The authors find a positive link between, on the one hand, increased perceived job productivity and, on the other hand, the number of hours working remotely before the lockdown and the degree of supervisor’s control. [45], on data from 700 teleworkers in Germany, highlight an increase in the perceived productivity and commitment during the lockdown. On UK data collected at five points in time (May, June, July, September, and November 2020, for the COVID-19 waves of ‘Understanding Society’), [58] show that increases in home office frequency are associated with a higher self-perceived productivity per hour. Using US data collected monthly between May and October 2020, [37] show that the home office experience has exceeded individuals’ expectations in terms of productivity for 61% of teleworkers, 26% estimate that their productivity is the same and 13% that it is lower.

Conversely, others studies conclude that home office induced by the lockdown periods has led mainly to a negative or an absence of job productivity evolution. By using data from Japan collected in June 2020, [46] shows a decrease of about 60% to 70%, compared to working at the usual workplace. [43] conclude to a decrease in subjective job performance during the lockdown compared to before by surveying 621 full-time workers. Using survey data collected internationally between 31^st^ March and 27^th^ April 2020 on 1,014 respondents, [47] show that 56% of respondents declared a lower productivity when they work from home compared to before, while 43% reported being at least as productive as before. [38], using data from the ‘Understanding Society’ COVID-19 survey carried out in UK, highlight that 28.9% of respondents said that they did more in June 2020 compared to 6 months before, 30.2% less and 40.9% the same (but when the home office practice is infrequent it reduces productivity). [59], based on data collected mostly in Quebec on 1,614 people in April 2020, report also that only one-third of respondents said they feel that their productivity has increased compared to before. [60], based on data collected from 704 academics at home office (between April and July 2020) show that the work efficiency for almost half of the researchers decreased during the lockdown compared to before while around a quarter of them were more efficient.

### 2.3 Moderating role of digital context

Some studies point out the importance of the digital context in the perceived evolution of well-being at work or job productivity during the lockdown. [49] show that the negative link between extensive use of home office and job satisfaction due to less interactions between co-workers is moderated by high-quality software. [61] underlines that videoconference has the role to maintain social ties and facilitate team work. Therefore, professional isolation risk to be more prevalent for disadvantaged employees in terms of digital skills who are at greater risk of diminished interactions [48, 62].

Some studies point out the importance of ICT knowledge and equipment in the perceived evolution of job productivity during the lockdown. For example, [59] reveal that having digital knowledge and being well equipped are important factors in improving productivity. Whereas, teleworkers who feel isolated and far from the decision-making process reported lower productivity. [63, 64] ran surveys during and after the lockdown on around 500 teleworkers. They observe that 37% declared that a lack of informal relationships and 38% that an information overload reduce their efficiency. [46] finds that the two main reasons of a decline of productivity when teleworking declared by employees are related to the lack of quick face-to-face interactions with colleagues (for 38.5% of the respondents) and a poor telecommunication environment at home relative to the workplace (for 34.9% of the respondents).

Nevertheless, the role played by the effective digital tool use on employees’ well-being and job productivity is, to the best of our knowledge, not yet studied in the framework of the COVID-19 lockdown periods. We can, thus, only rely on previous existing evidence that come from data collected before 2020 and most of the time that do not refer to the specific experience of teleworkers.

On the positive side, digital tools were shown to enhance communication and access to information through new tools and networks such as intranet, internal and external platforms at the workplace [65]. It can positively affect knowledge sharing which contribute to improving workers’ skills and human relations within teams and help reduce social isolation that can enhance job satisfaction and job productivity [66–68].

On the negative side, various drawbacks have been identified like the increase in time pressure, workload and of the permeability between the family and work spheres that can generate job stress [69–71].

Moreover, a potential misuse of digital tools may generate information overload or infobesity (too much information to have to deal with all the time) and be detrimental to employees. It was shown in a study conducted some months before the COVID-19 pandemic that the infobesity generates stress, and reduces job and life satisfaction [72]. Some studies focused on emails reveal that an intense use increase stress [73, 74].

Regarding teleworkers, few evidence, that come also from before 2020, exist and reveal positive impacts such as the enhance of workers’ sense of spatio-temporal flexibility, the reduce of social isolation and negative such as the sense that work is difficult to escape from [66, 75]. Nevertheless, the COVID-19 home office practice was digitally transformed [76]. It was shown that the diffusion of digital tools largely impacts the home office experienced by teleworkers before 2020 [77] but the consequences during the 2020 lockdown period remain largely unknown.

## 3 Data and methods

### 3.1 Data

We use data from the ‘COVID-19 Socio-Economic Impacts (SEI) survey’ conducted by LISER and the University of Luxembourg with the support of the Luxembourg National Research Fund (FNR). These data were collected between the end of May and the beginning of July 2020 with questions regarding before and during the lockdown induced by the COVID-19 health crisis in spring 2020.

As described in a report presenting the SEI survey [78], the data collection was organized as an open-ended online survey rather than a sample drawn using a probability sampling method. This allows for the collection of an initial rapid assessment of the socio-economic effects of COVID-19 on a sample of people who are easy to contact. The survey was announced through a press release and an extensive newspaper and social media campaign.

A comparison of the profile of the employed respondents with the profile of the whole working population of Luxembourg suggests an overrepresentation of women, residents, and employees working in the tertiary sector. A weighting procedure was used to ensure the representativeness of the studied population. The weights ensure that the distributions by gender, age (7 age classes: 20–29 years; 30–34; 35–39; 40–44; 45–49; 50–54; 55+), being a resident or a cross-border worker, and sector of activity of the employers (7 sectors: Primary/secondary/Trade/Horesca; Finance or insurance; Information and communication/professional, scientific, technical, administrative and support services; Public administration; Education; Human health and social work activities; Other services) are representative of people at work on the Luxembourgish labour market at 31 March 2020. The labour market figures of March 31, 2020 used to calibrate the sample come from the IGSS—Inspection Générale de la Sécurité Sociale (national social security institute) and were extracted online (https://adem.public.lu/fr/marche-emploi-luxembourg/faits-et-chiffres/statistiques/igss/Tableaux-interactifs-stock-emploi.html).

In the present analysis, we focus on employees who use the home office during the first lockdown of 2020. The studied sub-sample includes up to 438 employees who work for firms located in Luxembourg and are residents or cross-border employees. Due to the unavailability of official figures regarding the characteristics of teleworkers during the COVID-19, we used the weights calibrated on the whole employed population.

### 3.2 Variables

#### 3.2.1 Subjective well-being and job productivity

We measure teleworkers’ subjective well-being at work with job satisfaction and job stress. The measure of the evolution of job satisfaction is based on the following self-assessment question: *“Think about the overall satisfaction with your job*. *How would you rate it in the following periods*: *Before the lockdown (February 2020); During (early April 2020)*?*”*.

The measure of the evolution of job stress is based on the following self-assessment question: *“Please indicate your level of work-related stress in the following periods*: *Before the lockdown (February 2020); During (early April 2020)*?*”*. To evaluate the evolution of teleworkers’ subjective well-being we compare during and before the lockdown by subtracting the value of during with the value of before and we create two five-level variables corresponding to 1. Greatly decreased; 2. Decreased; 3. Remained the same; 4. Increased; 5 Greatly increased. In the robustness check of our results, we test a classification in three-level corresponding to 1. Decreased; 2. Remained the same; 3. Increased.

The measure of the evolution of job productivity comes from the following self-assessment question: *“Comparing your normal working conditions in February 2020 to those of April 2020*, *during the COVID-19 lockdown*, *would you say*: *April has been*: *1*. *Much less productive; 2*. *Less productive; 3*. *Just about as productive; 4*. *More productive; 5*. *Much more productive”*. As in other cross-section data analyses, the self-reported measures of employees’ productivity are not immune to measurement error. As stated by [79], when comparing data from different years or, in our case during a changing period like the lockdown period, the “effort norms were to increase, perhaps as a result of personal experience, of changing public attitudes to hard work or of media coverage of workplace stress, the changes in self-reported effort would be biased downward; this would strengthen [the] conclusions.” ([79], page 293). In addition, the reference group that employees think of when answering survey questions can influence their answers, as underlined in the job satisfaction literature [80].

#### 3.2.2 Digital tools use

Regarding the use of digital tools during the spring 2020 lockdown compared to before, we focus on four digital tools that enhance collaboration and strengthen communication between teleworkers that, at least partly compensate for the lack of face-to-face interactions: groupware that is a platform for collaborative work and documents sharing; workflow that are process automation tools; instant messaging; and web conference.

The data permits to capture the number of the four digital tools used, the growth of the use of these four tools between the lockdown and before and the frequency of use during the lockdown.

#### 3.2.3 Control variables

The control variables used are those commonly found in the literature focusing on well-being at work and job productivity [46, 81–84]. Thus, in our estimates of employee’s subjective well-being and job productivity, we control for a large scale of individual characteristics (e.g. age, gender, resident/cross border, level of education, household income, marital status, children, dwelling size in comparison to the number of inhabitants). We also control for the fact of having a previous experience of home office, the level of digital skills perceived before the spring 2020 lockdown, and for some worries encounter during the lockdown (about own heath, and about child(ren) school achievements when the respondent has child(ren) between 6 and 12 years old). We also consider job characteristics (e.g. full time or not, permanent contract or not, working hours evolution due to the lockdown, team work, managerial responsibilities, perceived degree of autonomy, perceived vulnerability on the labour market) and workplace characteristics (business sector, actions taken during the lockdown for the staff and for external partners). Descriptive statistics of all variables are presented in Table A1 in S1 Appendix.

### 3.3 Methods

First, we start by identifying profiles of teleworkers according to the evolution of their use of the four digital tools comparing before and during the lockdown and the frequency of use during. We focus on digital profiles and not on the use of a specific digital tool in order to take into account the effects of a cumulative use of digital tools on teleworkers’ well-being and productivity and the digital overload that could result [85–88]. In order to classify teleworkers usages, we use a Multiple Correspondence Analyses (MCA) followed by a hierarchical cluster analysis. In order to interpret the proximity between teleworkers’ digital tools use behavior, we introduce into our MCA, variables reflecting: the number of digital tools used before and during the lockdown (above or equal the median number of tools used), the evolution of use of each tool between before and during the lockdown (decline or stable use; extensive growth; intensive growth) and the frequency of use of each tool during the lockdown (less than weekly; weekly; daily). The socio-demographic characteristics do not intervene in this part of the analysis. As our data are quantitative, our cluster analysis is conducted on the coordinated computed by the MCA. We tested different specifications of our MCA, for instance, instead of using a variable capturing the evolution, we integrated the intensity of use of each tools before and during the lockdown. We used also a variable measuring the fact that teleworkers are below the median concerning the number of daily used tools instead of their position with the respect to the total number of tools used. We retained the specification with the most consistent criteria for choosing the number of profiles in the cluster analysis. This choice was based on the cubic clustering criterion, the semipartial R square, the pseudo F statistic, the pseudo t^2^ statistic and the dendogram [89, 90].

Second, we investigate the relationships between collaborative and communication digital tools profiles and the evolution of teleworkers’ job well-being (satisfaction and stress) and job productivity by comparing before and during the spring 2020 lockdown. Because our three measures have ordered values, we used ordered Probit models. The following equation was estimated for each of our three dependent variables:

$$Y_{d}=\alpha +\;\beta^{\prime }P+\;\delta^{\prime }X+\;\gamma^{\prime }J+\;\theta^{\prime }W+\;\varepsilon$$

The estimation of our two subjective well-being measures and our productivity measure (*Y*_1_ for *job satisfaction; Y*_2_ for *job stress; Y*_3_ for *job productivity)* equations includes the digital profiles (*P*), a constant (*α*); control variables about teleworkers’ characteristics (*X*); job characteristics (*J*) and workplace characteristics (*W*). ε represents a random error term normally distributed.

## 4 Results

### 4.1 Preliminary descriptive results

**Fig 1:**
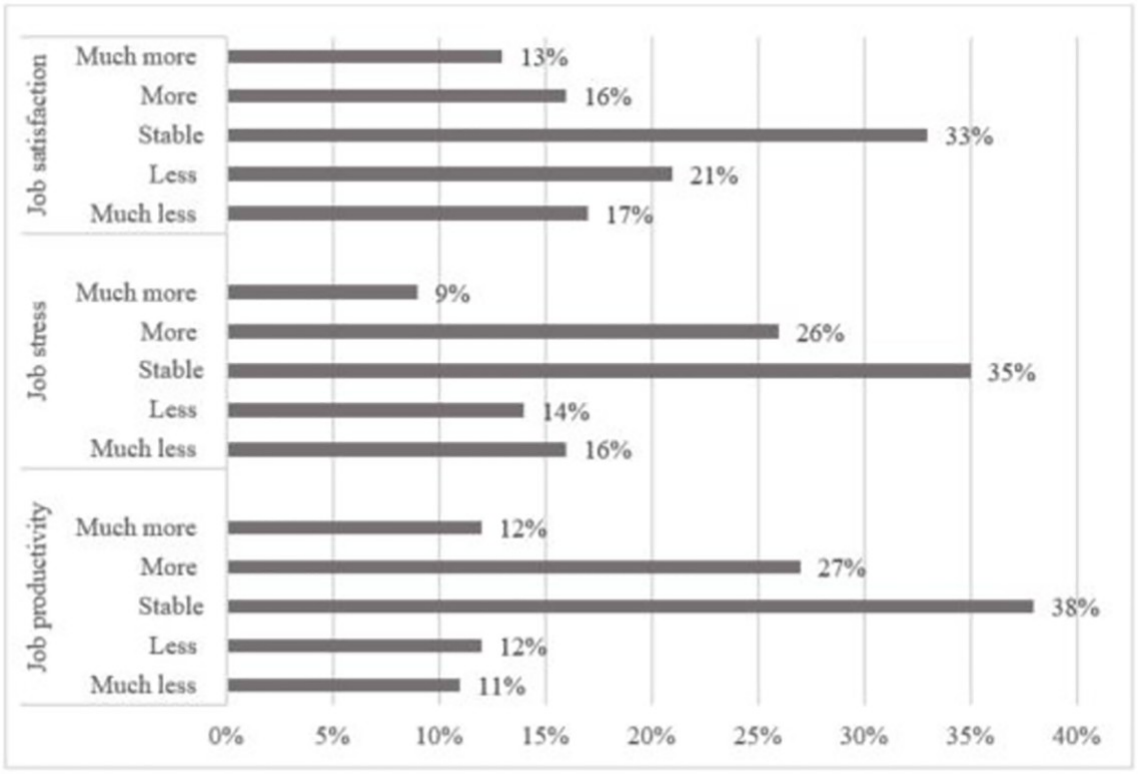
Descriptive statistics of the evolution of subjective well-being and productivity before and during the lockdown. *Source*: First wave of the Survey on the COVID-19 socio-economic impacts in Luxembourg (SEI), ‘home office’ module, LISER and University of Luxembourg. *Notes*: Weighted figures. The sub-sample used to study job stress covers 306 teleworkers, for the two others we have 438 observations.

The descriptive statistics underline a strong heterogeneity among teleworkers regarding the evolution of their well-being at work and job productivity due to the lockdown. These observations are consistent with others as [57] or [38] for job satisfaction or [59] for job productivity. Fig 1 reveals that the proportion of teleworkers who declared to be more satisfied by their job during the lockdown compared to before (29%) is significantly lower than the ones that are less satisfied (38%) but equivalent to those with a stable job satisfaction (33%).

We observe that the proportion of teleworkers who declared to be more stressed by their job during the lockdown compared to before (35%) is bigger but not significantly different from the ones that are less stressed (30%) and equivalent from those with a stable job stress (35%). Regarding job productivity, the Fig 1 shows that the proportion of teleworkers who declared to be less productive during the lockdown compared to before (23%) is significantly lower than the ones that are more productive (39%) and than the ones with a stable job productivity (38%).

As expected, teleworkers used more digital tools during the lockdown. In Table 1, the increase in the use of the four digital tools studied is underlined by the increase in the proportion of teleworkers who use at least three tools during the lockdown compared to before. This part rises from 59% to 74%.

**Table 1:** Use and intensity of use of digital tools.

		Mean
Equal 1 if the teleworker uses at least 3 digital tools (median) before the lockdown, 0 otherwise		59%
Equal 1 if the teleworker uses at least 3 digital tools during the lockdown, 0 otherwise		74%
Groupware use growth between before and during the lockdown	Decreased or remained the same	82%
	Extensive growth (discover)	8%
	Intensive growth	10%
Workflow growth between before and during the lockdown	Decreased or remained the same	76%
	Extensive growth (discover)	17%
	Intensive growth	7%
Instant messaging growth between before and during the lockdown	Decreased or remained the same	69%
	Extensive growth (discover)	16%
	Intensive growth	15%
Web conference growth between before and during the lockdown	Decreased or remained the same	55%
	Extensive growth (discover)	21%
	Intensive growth	24%
Groupware frequency of use during the lockdown	Less than weekly	32%
	Weekly	21%
	Daily	47%
Workflow frequency of use during the lockdown	Less than weekly	54%
	Weekly	15%
	Daily	31%
Instant messaging frequency of use during the lockdown	Less than weekly	21%
	Weekly	20%
	Daily	59%
Web conference frequency of use during the lockdown	Less than weekly	21%
	Weekly	31%
	Daily	48%

*Source*: First wave of the Survey on the COVID-19 socio-economic impacts in Luxembourg (SEI), ‘home office’ module, LISER and University of Luxembourg.

*Note*: Weighted figures.

In Table 1, we observe that the largest increase is for the use of web conference tools used to maintain eye contact with colleagues with 21% of teleworkers who discovered this tool and 24% who experienced an increase in their use intensity during the lockdown compared to before. The most frequently used digital tool during the lockdown period is the instant messaging, which is daily used by 59% of teleworkers.

### 4.2 Identification of five different profiles of teleworkers according to their collaborative and communication digital tools use

Five profiles emerge from the cluster analysis. Table 2 summarizes the main characteristics of each profile. Detailed descriptive statistics for each profile are presented in Table A2 in S1 Appendix.

**Table 2:** Characteristics of the different profiles.

Profile	Percentage of the sample	Average number of digital tools used before the lockdown	Average number of digital tools used during the lockdown	Extensive growth	Intensive growth	Use frequency during the lockdown
P1	31%	3.43	3.43	No	No	Daily
P2	18%	1.07	3.35	Yes	No	Weekly
P3	25%	1.32	1.27	No	No	Less than weekly
P4	18%	3.62	3.67	No	Yes	Daily
P5	8%	3.63	3.95	No	Yes (instant messaging & workflow)	Daily for instant messaging and weekly for the others tools

*Source*: First wave of the Survey on the COVID-19 socio-economic impacts in Luxembourg (SEI), ‘home office’ module, LISER and University of Luxembourg.

*Note*: Weighted figures.

The first profile encompasses 31% of teleworkers. The members of this profile used the same number of digital tools that favor online collaboration and communication during the lockdown as before (on average 3.4 out of 4 tools studied). During the lockdown, they did not experiment new tools or increase their frequency of use. They have a daily use of the four digital tools studied.

The second profile covers 18% of teleworkers. During the lockdown, they used a largest number of digital tools than before: 3.3 on average versus 1.1 before. 44% of the members of this group experiment groupware, 58% workflow or instant messaging and 70% web conference. They use digital tools experimented on a weekly basis and they continue to use digital tools used before also on this basis.

The third profile groups together 25% of teleworkers who did not use or had very limited use of digital tools that favor online collaboration and communication when face-to-face interactions are not possible. On average, both before and during the lockdown, they use 1.3 digital tools out of the four studied. They did not discover new digital tools during the lockdown or increase their frequency of use. When they used these tools, during the lockdown, it was less frequent than on a weekly basis.

The fourth profile covers 18% of teleworkers. Before and during the lockdown, they used many digital tools, on average 3.6 out of the four studied. While they did not experiment new digital tools during the lockdown, they increased their use of these tools. The majority of the members of this group used, during the lockdown, the digital tools on a daily basis. 60% used workflow on a daily basis (39% before the lockdown), 72% groupware (55% before the lockdown), 71% instant messaging (32% before the lockdown) and 94% web conference (20% before the lockdown).

The fifth profile concerns 8% of teleworkers. The number of digital tools used before and during the lockdown are above the median values as for the first and the fourth profiles. They are characterized by a stable use of groupware and an intensive growth of instant messaging and workflow. They have a weekly use of three tools except instant messaging that is used on a daily basis by 84% of members of this profile.

### 4.3 The link between collaborative and communication digital profiles and job well-being and job productivity

As shown by the main results reported in Table 3, the analysis highlights that the digital tools profiles are related to the evolution of job satisfaction, of job stress and of job productivity and that the relationships depend on the specific profiles.

**Table 3:** Collaborative and communication digital profiles and the evolution of subjective well-being and job productivity before and during the lockdown (ordered probit model).

	Evolution of job satisfaction	Evolution of job stress	Evolution of job productivity
	Coefficient	Coefficient	Coefficient
	(1)	(2)	(3)
P1 (stable and daily use)	-0.127 (0.174)	0.642*** (0.221)	-0.00209 (0.173)
P2 (extensive growth and weekly use)	-0.175 (0.190)	0.122 (0.231)	-0.0286 (0.189)
P3 (Non user/Limited user)	Ref.	Ref.	Ref.
P4 (intensive growth and daily use)	-0.844*** (0.202)	0.911*** (0.245)	-0.364* (0.202)
P5 (intensive growth limited to two digital tools and mainly weekly use)	-0.542** (0.270)	0.104 (0.382)	0.576** (0.271)
Individual characteristics	Yes	Yes	Yes
Job characteristics	Yes	Yes	Yes
Workplace characteristics	Yes	Yes	Yes
Observations	438	306	438
R-squared	0.1744	0.1818	0.1837

*Source*: First wave of the Survey on the COVID-19 socio-economic impacts in Luxembourg (SEI), ‘home office’ module, LISER and University of Luxembourg.

*Notes*: Weighted estimations. Standard errors in parentheses.

*Statistically significant at the 0.10 level;

** at the 0.05 level;

*** at the 0.01 level, ns not significant.

The results of the control variables are available in Table A3 in S1 Appendix.

For the first profile with a stable and a daily use of the four collaborative and communication digital tools, the result in Table 3 reveals that belonging to this profile increases job stress during the lockdown more than for the non-user/limited user profile (profile 3). For instance, belonging to the first profile, rather than the third one, increases the probability that the frequency of job stress has greatly increased by 0.083 conditional on the distribution of all the model variables being what they are in the dataset. Marginal effects are presented in Table A4 in S1 Appendix. A frequent use of such tools may generate too much information to deal with and the teleworkers may be subject to infobesity shown to be detrimental to employees’ well-being [72, 86]. Even if they had a daily use of these tools before the lockdown, the lockdown may have increased their stress due to, on the one hand, the impossibility of using non-digital means of communication and, on the other hand, the likely increase in the flow of information received (not measured in the data). Nevertheless, the evolution of their job satisfaction and job productivity do not differ from the non-user/limited user profile.

The information overload (or infobesity) is even more difficult to deal with for the members of profile fourth (intensive growth and daily use). This profile is, in fact, the one that is in the worst situation. Members of this group see both their job satisfaction and job productivity decrease during the lockdown and their job stress increase. The probability that their level of job satisfaction or job productivity has greatly increased, compared to the third profile, decreases respectively by 0.108 and 0.049, whereas the probability that its job stress has greatly increased raises by 0.137. In contrast to the first profile (stable and daily use), this profile has increased the intensity of use of digital tools. Members indeed did not use them on a daily basis before the lockdown while during the lockdown they use them daily. This means that they may have more difficulty than the members of the first profile in managing the flow of information received, as they are not used to face it.

The fifth profile (intensive growth limited to two digital tools and mainly weekly use) is positively related to an increase in job productivity (the probability that job productivity has greatly increased raises by 0.106) and negatively related to an increase in job satisfaction (the probability that job satisfaction has greatly increased decreases by 0.071). The fact that teleworkers with this profile used digital tools on a weekly basis (with the exception of instant messaging used on a daily basis) probably facilitates the management of information flows related to work (thus limiting the infobesity) and highlights the productivity gains of using such digital tools. This link is no found for the teleworkers with the second profile (extensive growth and weekly use) who have discovered digital tools during the lockdown. Therefore, teleworkers of the second profile may have had difficulty to exploit the full potential of digital tools. It seems that the infobesity trouble appears only when the use of the digital tools is not mastered and became difficult to manage especially when the use is on a daily basis as in the fourth profile (intensive growth and daily use). However, teleworkers’ productivity gains are at the expense of their job satisfaction. The use of digital tools, in the case of this fifth profile, seems to be mastered to be more performant in the job but not to maintain a high level of social interactions with co-workers that is necessary to improve job satisfaction as underlined, for instance, by [49].

Regarding the role played by control variables, Table A3 in S1 Appendix presents the full regression results.

Being a woman is negatively related to the growth of job satisfaction. The lockdown has intensified women’s domestic workload and the role conflict they faced [91] that may be detrimental to their job satisfaction. This result can also be explained by the gender differences in risk perception [92]. The gender is not significantly linked to the growth of job stress or job productivity.

The results show that only teleworkers aged between 40 and 49 are positively related to the growth of job satisfaction. Significant effect is not found for those aged between 30 and 39, or for those aged over 50. The findings are consistent with some studies suggesting that there is a parabolic relationship between home office satisfaction and age [93]. Proper remote working conditions at home and a stronger emphasis on social interactions [94] can explain the age different results observed between youngers and the others. Being 50 years of age or older is negatively related to the growth of job productivity suggesting that older workers would have more difficulty adapting to the organizational change imposed by the generalization of home office.

Previous experience of home office is negatively related to the growth of job satisfaction and positively related to the growth of job stress. This result, that differs from the one of [44], suggests that the working conditions at home were degraded during the lockdown compared to before. The calm time with less meetings and less interruptions obtained when not all employees worked at home, as shown by [26], seems to have disappeared during this period.

A moderate high level of digital skills (assessed by teleworkers regarding before the lockdown in comparison to a complex or advanced level) is positively linked to the growth of job satisfaction and negatively linked to the growth of job stress. This level of digital knowledge appears to be enough to help these teleworkers to evolve comfortably in a digitally transformed work environment. Having attended training (whatever the topic and length) during the lockdown is positively linked to the growth in job satisfaction and job productivity. Training courses enable employees to enrich their skills, which gives them additional resources to meet their new job demands.

An increase in work hours is, not highly surprisingly, positively related to job productivity [95]. Nevertheless, this increase in work hours is detrimental to teleworkers in terms of job stress whereas a decrease in work hours is negatively related to the growth of job stress. This result suggests that stressed teleworkers may need to work longer in order to cope with their workload that may also have increased especially if they had to replace absent co-workers who were sick or on leave to take care of their family members (such as managing homeschooling of children).

Regarding workplace characteristics, a stable time of team work is positively linked to the growth of both job satisfaction and job productivity (compared to less or more time of team work), reflecting the role that collaboration between co-workers can play to reduce social isolation when the work is performed at home [32].

Having stable managerial responsibilities is positively related to the growth of job satisfaction. An increase in managerial responsibilities is positively related to job productivity. This result suggests that teleworkers with a growth of their managerial responsibilities may feel that they are useful to their business and to support the virtual collaborative work of their team.

Regarding actions taken by the company during the lockdown, it appears that the intensification of relationships with employees’ representatives is positively linked to the growth of job productivity and negatively to the growth of job stress. As employees’ representatives act as guarantors of good working conditions and give voice to employees, strengthening the relations between upper management and employees’ representatives appear to be needed to have a performant workforce [96].

### 4.4 Effect heterogeneity

We investigate here further how different sub-groups of employees are affected: in particular, we differentiate employees by gender, age (20–29 years, 40 years and more), and education level (less than a master degree, at least a master degree).

#### 4.4.1 Evolution of job satisfaction

Exploratory sub-groups analyses reveal some differences. Regarding the belonging to the first profile (stable and daily use), the subsample of teleworkers with at least a master’s degree is negatively related to job satisfaction growth (Table 4). Even if highly educated teleworkers had a daily use of these tools before the lockdown, the lockdown may have increased the flow of information received. With more resort to communication tools, as underlined by [65], employees can easily ask more frequently experts to support them in their work, which increase the digital information flow and number of interruptions of high-skilled employees, negatively affecting their job satisfaction.

**Table 4:** Collaborative and communication digital profiles and the evolution of job satisfaction before and during the lockdown by sub-samples (ordered probit model).

	Whole sample	Men	Women	20–39 years	40 years and more	Less than a master degree	At least a master degree
	Coef.	Coef.	Coef.	Coef.	Coef.	Coef.	Coef.
P1 (stable and daily use) (Ref. P3 Non user/Limited user)	-0.127 (0.174)	-0.371 (0.284)	-0.242 (0.310)	0.470 (0.314)	-0.245 (0.269)	-0.165 (0.339)	-0.612** (0.257)
P2 (extensive growth and weekly use)	-0.175 (0.190)	-0.231 (0.301)	-0.180 (0.301)	0.658* (0.376)	-0.684*** (0.264)	-0.458 (0.325)	0.537* (0.303)
P4 (intensive growth and daily use)	-0.844*** (0.202)	-1.380*** (0.289)	-0.820** (0.352)	-0.221 (0.428)	-1.469*** (0.279)	-0.621 (0.394)	-0.825*** (0.317)
P5 (intensive growth limited to two digital tools and mainly weekly use)	-0.542** (0.270)	-1.612*** (0.395)	-0.209 (0.592)	-0.353 (0.659)	-0.0665 (0.412)	0.107 (0.516)	-0.847** (0.423)
Individual characteristics	Yes	Yes	Yes	Yes	Yes	Yes	Yes
Job characteristics	Yes	Yes	Yes	Yes	Yes	Yes	Yes
Workplace characteristics	Yes	Yes	Yes	Yes	Yes	Yes	Yes
Observations	438	136	302	165	273	212	226
R-squared	0.1744	0.3334	0.1713	0.338	0.2331	0.3137	0.2253

*Source*: First wave of the Survey on the COVID-19 socio-economic impacts in Luxembourg (SEI), ‘home office’ module, LISER and University of Luxembourg.

*Notes*: Weighted estimations. Standard errors in parentheses.

*Statistically significant at the 0.10 level;

** at the 0.05 level;

*** at the 0.01 level.

The absence of significant link between the second profile (extensive growth and weekly use) and the evolution of job satisfaction hides different links depending on the sub-sample studied. If a weekly use protects employees from too much notifications/interruptions, the discovery of new digital tools can be more challenging and more difficult to manage for some groups of workers counteracting the benefit of a weekly use. It seems particularly prevalent for the older workers. For them, belonging to the second profile is negatively link to the evolution of job satisfaction whereas a positive link appears for younger workers. This difference can be explained by the fact that cognitive skills are less malleable than non-cognitive skills at later ages [97] and learning ability declines with age [98]. The highly educated are better equipped to deal with the discovery of new tools than the others do and are thus more likely to benefit from a reasonable use of digital tools.

Belonging to the fourth profile (intensive growth and daily use) is negatively linked to the evolution of job satisfaction whatever the subsample studied. However, the link is not statically significant for younger employees and for the employees with less than a master degree.

Regarding the fifth profile (intensive growth limited to two digital tools and mainly weekly use), the results observed for the whole sample stay significant only for men and highly educated teleworkers.

#### 4.4.2 Evolution of job stress

Belonging to the first profile (stable and daily use) is positively linked to job stress evolution whatever the sub-sample studied. However, this relationship is only significant for the teleworkers who are at least 40 years old (Table 5). It suggests that the impossibility of using non-digital channel of communication during the lockdown is more detrimental to the mental health of older workers.

**Table 5:** Collaborative and communication digital profiles and the evolution of job stress before and during the lockdown by sub-samples (ordered probit model).

	Whole sample	Men	Women	20–39 years	40 years and more	Less than a master degree	At least a master degree
	Coef.	Coef.	Coef.	Coef.	Coef.	Coef.	Coef.
P1 (stable and daily use) (Ref. P3 Non user/Limited user)	0.642*** (0.221)	0.368 (0.440)	0.0469 (0.428)	0.273 (0.479)	1.298*** (0.341)	0.554 (0.525)	0.385 (0.321)
P2 (extensive growth and weekly use)	0.122 (0.231)	-0.393 (0.410)	0.578 (0.426)	0.839 (0.570)	0.558 (0.360)	1.063** (0.487)	-0.351 (0.380)
P4 (intensive growth and daily use)	0.911*** (0.245)	1.397***(0.407)	0.512 (0.451)	1.666*** (0.561)	1.313*** (0.367)	1.668*** (0.532)	0.723* (0.418)
P5 (intensive growth limited to two digital tools and mainly weekly use)	0.104 (0.382)	1.248 (0.865)	-0.260 (0.721)	-0.662 (1.187)	-0.477 (0.561)	-0.744 (0.662)	-0.809 (0.754)
Individual characteristics	Yes	Yes	Yes	Yes	Yes	Yes	Yes
Job characteristics	Yes	Yes	Yes	Yes	Yes	Yes	Yes
Workplace characteristics	Yes	Yes	Yes	Yes	Yes	Yes	Yes
Observations	306	95	211	115	191	137	169
R-squared	0.1818	0.4516	0.3079	0.3951	0.3061	0.3546	0.2529

*Source*: First wave of the Survey on the COVID-19 socio-economic impacts in Luxembourg (SEI), ‘home office’ module, LISER and University of Luxembourg.

*Notes*: Weighted estimations. Standard errors in parentheses.

*Statistically significant at the 0.10 level;

** at the 0.05 level;

*** at the 0.01 level.

Moving on to the belonging to the second profile (extensive growth and weekly use), it appears that the sub-sample of teleworkers with less than a Master’s degree is positively and significantly associated with a growth in job stress. The discovery of new tools may have been a source of stress for less educated teleworkers. Noted that for the highly educated this relationship is negative although not statistically significant.

Belonging to the fourth profile (intensive growth and daily use) is positively linked to the evolution of job stress whatever the subsample studied, however this link is not statistically significant for women.

For the fifth profile (intensive growth limited to two digital tools and mainly weekly use), no significant results appear whatever the subsample studied.

#### 4.4.3 Evolution of job productivity

For the belonging to the first profile (stable and daily use), no significant results appear whatever the subsample studied.

The analyses by sub-samples show a negative link between belonging to the second profile (extensive growth and weekly use) and the evolution of job productivity for teleworkers who are, at least, 40 years old (Table 6). The discovery of new tools may have led to a less favorable evolution of older teleworker’s productivity because they have more learning difficulties than younger people in line with the declines in cognitive skills malleability and learning ability with age underlined by [97, 98].

**Table 6:** Collaborative and communication digital profiles and the evolution of job productivity before and during the lockdown by sub-samples (ordered probit model).

	Whole sample	Men	Women	20–39 years	40 years and more	Less than a master degree	At least a master degree
	Coef.	Coef.	Coef.	Coef.	Coef.	Coef.	Coef.
P1 (stable and daily use) (Ref. P3 Non user/Limited user)	-0.002 (0.173)	0.095 (0.277)	0.060 (0.311)	0.226 (0.305)	0.077 (0.269)	-0.009 (0.334)	-0.125 (0.262)
P2 (extensive growth and weekly use)	-0.0286 (0.189)	-0.448 (0.305)	0.111 (0.299)	0.353 (0.375)	-0.471* (0.261)	0.003 (0.318)	0.153 (0.305)
P4 (intensive growth and daily use)	-0.364* (0.202)	-0.743*** (0.286)	0.065 (0.350)	-0.393 (0.411)	-0.478* (0.272)	-0.376 (0.378)	-0.540 (0.330)
P5 (intensive growth limited to two digital tools and mainly weekly use)	0.576** (0.271)	0.124 (0.374)	0.978 (0.621)	-0.005 (0.586)	0.513 (0.421)	0.798 (0.540)	0.756* (0.419)
Individual characteristics	Yes	Yes	Yes	Yes	Yes	Yes	Yes
Job characteristics	Yes	Yes	Yes	Yes	Yes	Yes	Yes
Workplace characteristics	Yes	Yes	Yes	Yes	Yes	Yes	Yes
Observations	438	136	302	165	273	212	226
R-squared	0.1837	0.3173	0.1780	0.3163	0.2284	0.2801	0.2638

*Source*: First wave of the Survey on the COVID-19 socio-economic impacts in Luxembourg (SEI), ‘home office’ module, LISER and University of Luxembourg.

*Notes*: Weighted estimations. Standard errors in parentheses.

*Statistically significant at the 0.10 level;

** at the 0.05 level;

*** at the 0.01 level.

Except for women, belonging to the fourth profile (intensive growth and daily use) is negatively linked to the evolution of job productivity. However, this link remains statistically significant only for men and teleworkers who are, at least, 40 years old.

The positive link observed in the whole sample between the fifth profile (intensive growth limited to two digital tools and mainly weekly use) and the evolution of job productivity is only statistically significant for highly educated teleworkers.

### 4.5 Robustness checks

For the robustness checks of our results, we test a different breakdown of our dependent variables into three categories (decrease, stability, increase) instead of five. The links observed between the teleworkers’ collaborative and communication digital profiles and the evolution of their job satisfaction, job stress and job productivity remain except for fifth profile (intensive growth limited at two digital tools and mainly weekly use) where the link is no more significant for the evolution of job satisfaction and only significant at the 12% threshold for the evolution of productivity evolution. We also perform an ordered Logit model. The results obtain for our variables of interest are similar to those obtained from the ordered Probit model.

## 5 Conclusion

The COVID-19 induced lockdown periods have shaken employees’ relationship to work, in time, space and form, for an important part of the working population [8–10]. This place to work change resulted in a soar of digital tool uses to overcome the lack of face-to-face interactions to collaborate and perform work outside the office as underlined both by services providers (Zoom, Microsoft) and scholars [76]. Existing research on consequences of home office on employees’ job well-being and job productivity, during the lockdowns, are heterogeneous and mixed [38, 43]. Moreover, even if the importance of a well-equipped digital work environment is acknowledged by various analyses [46, 49, 59], the role of the effective use of collaborative and communication digital tools during the lockdown periods on teleworkers’ job well-being and job productivity remains largely unknown.

Our analysis, is one of the first, that provides an assessment of how the uses of collaborative and communication digital tools are related to the evolution of teleworkers’ job satisfaction, job stress and job productivity comparing during and before the first lockdown in spring 2020. We answer these questions through a unique survey conducted in May-July 2020 on employees working in Luxembourg. The main contribution of the paper to the literature is to broaden the empirical evidence regarding the digitally transformed home office.

Our results provide, first, five profiles regarding the evolution of usage of four collaborative and communication digital tools (groupware, workflow, instant messaging and web conference) during the lockdown compared to before and the frequency of each tool usage during the lockdown. Second, these five digital profiles are related to the evolution of job satisfaction, job stress and job productivity. More specifically, three main results emerged.

First, the profile that generates the higher growth of job productivity is the one with a combined mastered and job performance-oriented strategy of use of all of the four collaborative and communication digital tools on a daily or weekly basis. Nevertheless, teleworkers’ productivity gains are at the expense of their job satisfaction mainly due to the lack of a social interaction-oriented strategy of use [49]. Second, on the contrary, the use of the four studied collaborative and communication digital tools during the lockdown, associated with an increase in the frequency of use, seems to generate too much information flow to deal with and the teleworkers may be subject to information overload that increases their job stress and reduces their job satisfaction and job productivity [85, 86]. Third, the habit of using the four digital tools on a daily basis already before the lockdown appears to protect teleworkers from most of the adverse effects, except for an increase in their job stress during the lockdown.

Our results underline the importance of tackling the impending digital skills lack among different groups of employees. The discovery of new digital tools seems to have been more difficult to use for older and less educated teleworkers. Indeed, for these teleworkers, the discovery of digital tools has cancelled out the positive effect of a limited use on the growth of job satisfaction (for older workers) or on the decrease in the frequency of job stress (for the less educated).

Our results have practical implications at many levels. For managers, the literature have already identified practical solutions to tackle the drawbacks of digital tool use, such as the importance of boundaries management [99, 100] as well as the control of digital interruptions [74]. In consequence, good practices related to the use of digital tools and especially to the management of notifications and interruptions, that broke employees’ concentration, need to be spread by managers to the staff in order to overcome the detrimental effects on job well-being and job productivity. Anyway, the need for a proximity management towards the employees is accentuated by the COVID-19 crisis. For their well-being and productivity, links allowing proximity, closeness and avoiding teleworkers isolation are necessary. These links can be supported by digital tools.

For the employees, a better knowledge of the various profiles of use of the different digital tools allows to better ensure their appropriation. Our analyses allow a better knowledge of the affordances of digital tools in terms of communication and collaboration. Employees are then supported in their choice of combination between these digital tools, considering the evolution of the COVID-19 crisis.

For companies adopting these digital solutions, a better understanding of usage profiles in times of long-term crisis is an asset in designing a strategy for the evolution of the digital tools’ adoption. A specific training strategy should be implemented for both teleworkers and managers to improve the digital skills required to be familiar of the new digital tools and the digital information flow.

Additional practical policy conclusions that stem from the results should pay attention to legal rules applied in the country. Indeed, employees need to be communicated on the aspects related to telework law and arrangements. For instance, in France, teleworking and teleworkers are framed by laws (e.g. law of 22 march 2012) and arrangements (e.g. A.N.I arrangements of 2 April 2021). In Luxembourg, an inter-branch agreement regulates telework since 2006. A new telework agreement signed in October 2020 replaced the 2006 one and came into force on February 2, 2021. The right to disconnect is an example of solution to limit flow of information, communication and work after regular work hours. The right to disconnect emerged in France, but other countries (or cities) are ready to adopt such law. For instance, the New York City Council proposed in January 2019 a local law for private employees disconnecting from electronic communications during non-work hours. The French government adopted a law “Adapting the Labour Law to the Digital Age” (Article L. 2242–8 of the French Labour Code) and the article 55(1) entered into force on January 1, 2017 includes the right to disconnect.

Our study is confronted to some limits. First, the data used do not give information on the actual flow of information received by teleworkers. Second, the data do not give information on the quality of digital equipment of teleworkers which can play a role on their well-being and productivity evolution due to the lockdown. Third, countries other than Luxembourg may show different results, for various reasons including socio-economic, legal or cultural dimensions. These topics should be the focus of future research.

## Supporting information

S1 Appendix (DOCX)
