# Supplementary material for: Digitally transformed home office impacts on job satisfaction, job stress and job productivity. COVID-19 findings
Source: PLoS One. 2022 Mar 10;17(3):e0265131. doi: 10.1371/journal.pone.0265131 (PMC8912217; doi:10.1371/journal.pone.0265131)
Supplement: S1 Appendix — (DOCX) [file pone.0265131.s001.docx]

#### Table A1. Descriptive statistics

|  | Mean | Std. Dev. |
| --- | --- | --- |
| Evolution of job satisfaction | 2.886 | 1.251 |
| Evolution of job stress | 2.975 | 1.178 |
| Evolution of job productivity | 3.171 | 1.137 |
| P1 (stable and daily use) (Ref. P3 Non user/Limited user) | 0.308 |  |
| P2 (extensive growth and weekly use) | 0.184 |  |
| P4 (intensive growth and daily use) | 0.180 |  |
| P5 (intensive growth limited to two digital tools and mainly weekly use) | 0.076 |  |
| *Individual characteristics* |  |  |
| Men | 0.629 |  |
| 30-39 years (Ref. 20-29 years) | 0.247 |  |
| 40-49 years | 0.320 |  |
| 50+ years | 0.231 |  |
| Resident (ref. cross-border workers) | 0.521 |  |
| Large surface per inhabitant in the dwelling | 0.505 |  |
| Alone without child(ren) below 12 years old (Ref. in couple with a partner not working during the lockdown with or without child(ren) | 0.207 |  |
| Alone or in couple with a partner working during the lockdown without child(ren) below 12 years old | 0.313 |  |
| In couple with a partner working during the lockdown without child(ren) below 12 years old | 0.087 |  |
| Alone or in couple with child(ren) between 7 and 12 years old and being worried about their school achievement | 0.241 |  |
| Highly concerned about his/her health | 0.325 |  |
| Tertiary education below Master (Ref. Primary or secondary) | 0.286 |  |
| Master, Doctoral level or equivalent | 0.547 |  |
| Household income 4,000 - 6,000 euros (Ref. less than 4.000€) | 0.353 |  |
| 6,000 - 8,000 euros | 0.277 |  |
| Greater than 8,000 euros | 0.228 |  |
| IT skills level – Basic or moderate low (Ref. Complex/advanced) | 0.236 |  |
| Moderate medium | 0.183 |  |
| Moderate high | 0.222 |  |
| Take online training(s) during the lockdown | 0.254 |  |
| Previous experience of telework | 0.174 |  |
| *Job characteristics* |  |  |
| Permanent contract | 0.899 |  |
| Part-time | 0.133 |  |
| Risk of losing the job in the next 6 months (1-7) | 1.917 | 1.389 |
| Easiness to find a job with a similar salary (1-7) | 3.914 | 1.906 |
| Decrease in working hours during the lockdown (Ref. Remained the same) | 0.175 |  |
| Increase in working hours during the lockdown | 0.155 |  |
| Decrease in atypical working hours (Ref. Remained the same) | 0.084 |  |
| Increase in atypical working hours during the lockdown | 0.198 |  |
| Team work - remained the same (Ref. decreased) | 0.654 |  |
| Team work - increased | 0.075 |  |
| Having managerial responsibilities - remained the same (Ref. decreased) | 0.835 |  |
| Having managerial responsibilities - increased | 0.067 |  |
| Autonomy (z-score of the sum of the items) (a) | 0.090 | 1.040 |
| *Workplace characteristics* |  |  |
| Firm intensify its relationships with employees (z-score) | 0.230 | 0.956 |
| Firm intensify its relationships with employees’ representatives (z-score) | 0.116 | 1.053 |
| Firm set up work arrangements that go beyond legal obligations (z-score) | 0.069 | 0.984 |
| Internal information sharing (z-score of the sum of the items) (b) | 0.009 | 1.036 |
| Working for a firm that set up external actions (z-score of the sum) (c) | 0.106 | 0.924 |
| Finance and insurance sector (Ref. primary, secondary, trade, horesca sectors) | 0.159 |  |
| Non-financial service sector | 0.317 |  |
| Public administration and education | 0.155 |  |
| Human health and social work activities | 0.084 |  |
| Observations | 438* |  |

*Source*: First wave of the Survey on the COVID-19 socio-economic impacts in Luxembourg (SEI), ‘Work from home’ module, LISER and University of Luxembourg. Weighted figures; Standard deviations are only reported for non-binary variables.

*Notes*:

* 306 observations in the case of the evolution of job stress.

(a) The included items to measure autonomy are the following: Autonomy in work organization and methods; Learn from mistakes, without being penalized; Strongly empowered in own work and results.

(b) The included items to measure the internal information sharing are: Having access to full information useful for work; Informed of the firms' strategic priorities; Manager’s support; Communication with colleagues without having to respect the hierarchy.

(c) The included items to measure firm’s external actions are the following: Firm set up actions for civil society; Firm set up actions for its suppliers, customers and subcontractors; Firm set proactive actions without waiting to be called upon.

#### Table A2. Cluster analysis

|  |  | Mean percentage | | | | | |
| --- | --- | --- | --- | --- | --- | --- | --- |
|  |  | **P1** | **P2** | **P3** | **P4** | **P5** | **All sample** |
| Number of tools used before the lockdown | Use less than 3 tools | 3.5% | **94%** | **88%** | .08% | 5.9% | 41% |
|  | Use at least 3 tools | **96%** | 6.1% | 12% | **100%** | **94%** | 59% |
| Number of tools used during the lockdown | Use less than 3 tools | 2.5% | .00% | **100%** | .00% | .00% | 26% |
|  | Use at least 3 tools | **97%** | **100%** | .00% | **100%** | **100%** | 74% |
| Groupware use growth between before and during the lockdown | Decreased or remained the same | **93%** | 48% | **99%** | 67% | 91% | 82% |
|  | Extensive growth | .00% | **44%** | .89% | .00% | 2.0% | 8% |
|  | Intensive growth | 6.7% | 7.7% | .00% | **33%** | 7.0% | 10% |
| Workflow growth between before and during the lockdown | Decreased or remained the same | **86%** | 42% | **99%** | 68% | 66% | 76% |
|  | Extensive growth | 12% | **58%** | 1.3% | 2.9% | 22% | 17% |
|  | Intensive growth | 2.0% | .04% | .00% | **29%** | **12%** | 7% |
| Instant messaging growth between before and during the lockdown | Decreased or remained the same | **100%** | 26% | 76% | 58% | 47% | 69% |
|  | Extensive growth | .00% | **58%** | 15% | 7.1% | .00% | 16% |
|  | Intensive growth | .00% | 17% | 8.8% | **35%** | **53%** | 15% |
| Web conference growth between before and during the lockdown | Decreased or remained the same | **88%** | 14% | **65%** | 23% | 60% | 55% |
|  | Extensive growth | 6.2% | **70%** | 22% | 2.0% | 8.8% | 21% |
|  | Intensive growth | 5.8% | 16% | 14% | **75%** | 31% | 24% |
| Groupware frequency of use during the lockdown | Less than weekly | 11% | 39% | **85%** | 1.0% | 4.7% | 32% |
|  | Weekly | 17% | 23% | 7.0% | 26% | **60%** | 21% |
|  | Daily | **71%** | 39% | 8.4% | **72%** | 36% | 47% |
| Workflow frequency of use during the lockdown | Less than weekly | 47% | 49% | **97%** | 33% | .00% | 54% |
|  | Weekly | 6.8% | **25%** | .00% | 6.9% | **94%** | 15% |
|  | Daily | **46%** | 26% | 2.7% | **60%** | 6.4% | 31% |
| Instant messaging frequency of use during the lockdown | Less than weekly | 12% | 11% | **60%** | 3.6% | 1.0% | 21% |
|  | Weekly | 8.1% | **41%** | 17% | 25% | 15% | 20% |
|  | Daily | **80%** | 48% | 23% | **71%** | **84%** | 59% |
| Web conference frequency of use during the lockdown | Less than weekly | 18% | 18% | **48%** | .32% | .00% | 21% |
|  | Weekly | 22% | **55%** | 22% | 5.8% | **97%** | 31% |
|  | Daily | **60%** | 27% | 30% | **94%** | 3.3% | 48% |

*Source*: First wave of the Survey on the COVID-19 socio-economic impacts in Luxembourg (SEI), ‘Work from home’ module, LISER

and University of Luxembourg.

*Note*: Weighted figures.

#### Table A3. Full regressions results of Table 3

|  | Evolution of job satisfaction | Evolution of job stress | Evolution of job productivity |
| --- | --- | --- | --- |
|  | Coef. | Coef. | Coef. |
|  | (1) | (2) | (3) |
| P1 (stable and daily use) (Ref. P3 Non user/Limited user) | -0.127 | 0.642*** | -0.00209 |
|  | (0.174) | (0.221) | (0.173) |
| P2 (extensive growth and weekly use) | -0.175 | 0.122 | -0.0286 |
|  | (0.190) | (0.231) | (0.189) |
| P4 (intensive growth and daily use) | -0.844*** | 0.911*** | -0.364* |
|  | (0.202) | (0.245) | (0.202) |
| P5 (intensive growth limited to two digital tools and mainly weekly use) | -0.542** | 0.104 | 0.576** |
|  | (0.270) | (0.382) | (0.271) |
| Men | 0.353** | 0.0336 | 0.000625 |
|  | (0.147) | (0.190) | (0.146) |
| 30-39 years (Ref. 20-29 years) | 0.0443 | 0.0780 | -0.327 |
|  | (0.225) | (0.283) | (0.221) |
| 40-49 years | 0.705*** | -0.157 | 0.220 |
|  | (0.222) | (0.280) | (0.218) |
| 50+ years | -0.145 | 0.277 | -0.467* |
|  | (0.236) | (0.295) | (0.239) |
| Resident (ref. cross-border workers) | -0.330** | 0.0785 | -0.402*** |
|  | (0.154) | (0.192) | (0.155) |
| Large surface per inhabitant in the dwelling | -0.106 | -0.290 | 0.156 |
|  | (0.141) | (0.184) | (0.143) |
| Alone without child(ren) below 12 years old (Ref. in couple with a partner not working during the lockdown with or without child(ren) | 0.472** | -0.132 | 0.119 |
|  | (0.238) | (0.297) | (0.239) |
| Alone or in couple with a partner working during the lockdown without child(ren) below 12 years old | 0.306* | -0.421* | 0.130 |
|  | (0.174) | (0.218) | (0.172) |
| In couple with a partner who didn’t work during the lockdown without child(ren) below 12 years old | -0.0720 | 0.343 | 0.582** |
|  | (0.262) | (0.313) | (0.266) |
| Alone or in couple with child(ren) between 7 and 12 years old and being worried about their school achievement | 0.165 | -0.263 | 0.214 |
|  | (0.163) | (0.207) | (0.165) |
| Highly concerned about his/her health | -0.217 | 0.334* | 0.0957 |
|  | (0.139) | (0.178) | (0.139) |
| Tertiary education below Master (Ref. Primary or secondary) | -0.330 | -0.141 | -0.179 |
|  | (0.205) | (0.282) | (0.205) |
| Master, Doctoral level or equivalent | -0.326* | -0.0885 | -0.271 |
|  | (0.190) | (0.266) | (0.191) |
| Household income 4,000 - 6,000 euros (Ref. less than 4.000€) | 0.527** | -0.181 | 0.279 |
|  | (0.219) | (0.274) | (0.222) |
| 6,000 - 8,000 euros | 0.222 | -0.160 | 0.170 |
|  | (0.246) | (0.306) | (0.248) |
| Greater than 8,000 euros | -0.300 | 0.109 | -0.0311 |
|  | (0.284) | (0.373) | (0.288) |
| IT skills level – Basic or moderate low (Ref. Complex/advanced) | -0.105 | -0.232 | -0.166 |
|  | (0.187) | (0.236) | (0.190) |
| Moderate medium | -0.113 | 0.0112 | -0.325* |
|  | (0.190) | (0.235) | (0.191) |
| Moderate high | 0.410** | -0.443** | 0.0704 |
|  | (0.180) | (0.223) | (0.181) |
| Take online training(s) during the lockdown | 0.562*** | -0.283 | 0.529*** |
|  | (0.157) | (0.195) | (0.157) |
| Previous experience of telework | -0.451*** | 0.484** | -0.0115 |
|  | (0.157) | (0.201) | (0.159) |
| Permanent contract | 0.344 | -0.954*** | 0.795*** |
|  | (0.222) | (0.267) | (0.227) |
| Part-time | 0.0926 | 0.0516 | 0.209 |
|  | (0.203) | (0.268) | (0.205) |
| Risk of losing the job in the next 6 months | -0.0643 | -0.0243 | -0.0315 |
|  | (0.0485) | (0.0581) | (0.0484) |
| Easiness to find a job with a similar salary | -0.00590 | -0.0340 | -0.0784** |
|  | (0.0353) | (0.0413) | (0.0358) |
| Decrease in working hours during the lockdown (Ref. Remained the same) | 0.183 | -0.950*** | -0.387** |
|  | (0.174) | (0.226) | (0.175) |
| Increase in working hours during the lockdown | -0.161 | 0.743*** | 0.814*** |
|  | (0.190) | (0.256) | (0.194) |
| Decrease in atypical working hours (Ref. Remained the same) | 0.388 | -0.361 | 0.0864 |
|  | (0.247) | (0.297) | (0.240) |
| Increase in atypical working hours during the lockdown | 0.232 | 0.0789 | -0.146 |
|  | (0.164) | (0.207) | (0.165) |
| Team work - remained the same (Ref. decreased) | 0.594*** | -0.117 | 0.656*** |
|  | (0.156) | (0.192) | (0.157) |
| Team work - increased | 0.394 | -0.339 | 0.101 |
|  | (0.283) | (0.359) | (0.284) |
| Having managerial responsibilities - remained the same (Ref. decreased) | 0.398* | -0.427 | 0.194 |
|  | (0.239) | (0.294) | (0.238) |
| Having managerial responsibilities - increased | 0.108 | 0.526 | 1.026*** |
|  | (0.350) | (0.472) | (0.362) |
| Autonomy (z-score of the sum of the items) | 0.433*** | -0.167* | 0.356*** |
|  | (0.0705) | (0.0890) | (0.0694) |
| Firm intensify its relationships with employees | -0.0742 | -0.123 | -0.112 |
|  | (0.0885) | (0.107) | (0.0885) |
| Firm intensify its relationships with employees’ representatives | 0.0220 | -0.171* | 0.285*** |
|  | (0.0743) | (0.0974) | (0.0760) |
| Firm set up work arrangements that go beyond legal obligations | 0.116 | 0.00145 | 0.106 |
|  | (0.0732) | (0.0914) | (0.0733) |
| Internal information sharing (z-score of the sum of the items) | 0.0635 | 0.0281 | -0.284*** |
|  | (0.0756) | (0.0965) | (0.0771) |
| Working for a firm that set up external actions (z-score of the sum) | -0.00480 | 0.135 | -0.220*** |
|  | (0.0811) | (0.103) | (0.0821) |
| Finance and insurance sector (Ref. primary, secondary, trade, horesca sectors) | 0.0208 | 0.976*** | 0.0403 |
|  | (0.209) | (0.282) | (0.210) |
| Non-financial service sector | 0.375** | 0.474** | 0.191 |
|  | (0.179) | (0.237) | (0.178) |
| Public administration and education | 0.418* | 0.289 | 0.0460 |
|  | (0.220) | (0.303) | (0.222) |
| Human health and social work activities | 0.0753 | 0.578* | -0.0331 |
|  | (0.253) | (0.335) | (0.254) |
| Observations | 438 | 306 | 438 |
| R-squared | 0.1744 | 0.1818 | 0.1837 |

*Source*: First wave of the Survey on the COVID-19 socio-economic impacts in Luxembourg (SEI), ‘Work from home’ module, LISER and University of Luxembourg.

*Notes*: Weighted estimations. Standard errors in parentheses. *Statistically significant at the 0.10 level; ** at the 0.05 level; *** at the 0.01 level.

#### Table A4. Marginal effects regarding the magnitude of higher value of the employees’ outcomes studied (based on the full regressions)

|  | **Evolution of job satisfaction** | **Evolution of job stress** | **Evolution of job productivity** |
| --- | --- | --- | --- |
|  | (1) | (2) | (3) |
| P1 (stable and daily use) | -0.020  (0.026) | **0.083****  (0.033) | -0.000  (0.026) |
| P2 (extensive growth and weekly use) | -0.026  (0.028) | 0.014  (0.029) | -0.004  (0.027) |
| P3 (Non user/Limited user) | Ref. | Ref. | Ref. |
| P4 (intensive growth and daily use) | **-0.108*****  (0.021) | **0.137*****  (0.047) | **-0.049****  (0.025) |
| P5 (intensive growth limited to two digital tools and mainly weekly use) | **-0.071****  (0.028) | 0.012  (0.048) | **0.106***  (0.059) |
| Individual characteristics | Yes | Yes | Yes |
| Job characteristics | Yes | Yes | Yes |
| Workplace characteristics | Yes | Yes | Yes |
| Observations | 438 | 306 | 438 |

*Source*: First wave of the Survey on the COVID-19 socio-economic impacts in Luxembourg (SEI), ‘Work from home’ module, LISER and University of Luxembourg.

*Notes*: Weighted estimations. Marginal effect; standard errors in parentheses. *Statistically significant at the 0.10 level; ** at the 0.05 level; *** at the 0.01 level.
